# FRET‐FLIM to Determine Protein Interactions and Membrane Topology of Enzyme Complexes

**DOI:** 10.1002/cpz1.598

**Published:** 2022-10-27

**Authors:** Tatiana Spatola Rossi, Charlotte Pain, Stanley W. Botchway, Verena Kriechbaumer

**Affiliations:** ^1^ Endomembrane Structure and Function Research Group, Biological and Medical Sciences Oxford Brookes University Oxford UK; ^2^ Central Laser Facility, Science and Technology Facilities Council (STFC) Rutherford Appleton Laboratory Research Complex at Harwell Didcot UK

**Keywords:** eGFP, endoplasmic reticulum, FRET‐FLIM, membrane protein, mRFP, protein‐protein interactions, topology

## Abstract

Determining protein‐protein interactions is vital for gaining knowledge on cellular and metabolic processes including enzyme complexes and metabolons. Förster resonance energy transfer with fluorescence lifetime imaging microscopy (FRET‐FLIM) is an advanced imaging methodology that allows for the quantitative detection of protein‐protein interactions. In this method, proteins of interest for interaction studies are fused to different fluorophores such as enhanced green fluorescent protein (eGFP; donor molecule) and monomeric red fluorescent protein (mRFP; acceptor molecule). Energy transfer between the two fluorophore groups can only occur efficiently when the proteins of interest are in close physical proximity, around ≤10 nm, and therefore are most likely interacting. FRET‐FLIM measures the decrease in excited‐state lifetime of the donor fluorophore (eGFP) with and without the presence of the acceptor (mRFP) and can therefore give information on protein‐protein interactions and the membrane topology of the tested protein. Here we describe the production of fluorescent protein fusions for FRET‐FLIM analysis in tobacco leaf epidermal cells using *Agrobacterium*‐mediated plant transformation and a FRET‐FLIM data acquisition and analysis protocol in plant cells. These protocols are applicable and can be adapted for both membrane and soluble proteins in different cellular localizations. © 2022 The Authors. Current Protocols published by Wiley Periodicals LLC.

**Basic Protocol 1**: Protein expression in tobacco leaf cells via transient *Agrobacterium*‐mediated plant transformation

**Basic Protocol 2**: FRET‐FLIM data acquisition and analysis

## INTRODUCTION

**Figure 1:**
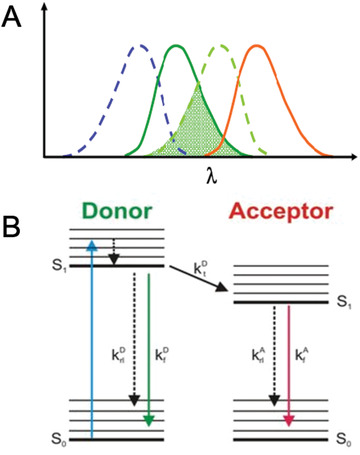
Förster resonance energy transfer (FRET) diagram. (**A**) Illustration of relationship between donor and acceptor spectra. Donor absorbance is shown as a blue dotted line, with donor emission in green; acceptor absorbance is shown as a green dotted line, with acceptor emission in red. (**B**) Energy diagram describing energy transfer via the FRET mechanism from donor to acceptor.

Enzyme complexes are an integral part of the cell and its metabolic processes. In biotechnology applications, testing protein‐protein interactions is required to assay correct complex formation, which enables correct biological functions. In the case of integral membrane complexes, membrane topology (the orientation of proteins in a membrane) is also key to evaluating correct complex assembly. One method used to determine protein‐protein interactions is Förster resonance energy transfer (FRET), which is based on the nonradiative transfer of energy between two fluorophores found in close proximity and can measure interactions with subcellular specificity *in vivo* (Chen, Mills, & Periasamy, 2003). For FRET to occur, the fluorophores must have significant spectral overlap; that is, the emission spectrum of a “donor” fluorophore must overlap with the absorption spectrum of an “acceptor” fluorophore for energy to be transferred from the donor to the acceptor (Wallrabe & Periasamy, 2005; Fig. 1).

FRET efficiency is inversely proportional to the sixth power of the distance between fluorophores and therefore is only detectable when the proteins are found at <10 nm from each other. Energy transfer can be calculated with the equation:

$$E\;=\;\frac{1}{{\left(1\;+\;\left[\frac{r}{R_{0}}\right]\right)}^{6}}$$

where r is the donor and acceptor chromophore separation distance and R_0_ is the Förster radius with 50% transfer efficiency.

The florescence lifetime is a rate reaction so that:

$$\tau \;=\;\frac{1}{\left(k_{r}\;+\;k_{\mathrm{nr}}\right)}$$

where k is the rate and r and nr are radiative and nonradiative processes, respectively.

The energy transfer occurs, and the excited‐state lifetime is quenched. E can therefore be calculated as:

$$E\;=\;1\;-\;\left(\frac{T_{\mathrm{DA}}}{T_{D}}\right)$$

or as a percentage:

$$E\%\;=\;\left(1\;-\;\left[\frac{I_{\mathrm{DA}}}{I_{D}}\right]\right)\;\times \;100$$

where τ_DA_ is the lifetime of the donor in the presence of the acceptor and τ_D_ is the lifetime of the donor in the absence of acceptor.

FRET efficiency is also dependent on the quantum yield of the donor and the alignment of the transition dipole‐dipole moments of the fluorophores (Corry, Jayatilaka, Martinac, & Rigby, 2006). The transfer of energy from donor to acceptor causes quenching of the donor fluorescence, which is measured in intensity‐based (or spectral) FRET.

In a biological system, the proteins of interest are fused to donor and acceptor fluorophores: enhanced green fluorescent protein (eGFP; with absorbance at 488 nm and emission at 510 nm; Cormack, Valdivia, & Falkow, 1996) and monomeric red fluorescent protein (mRFP; with absorbance at 584 nm and emission at 607 nm; Campbell et al., 2002), respectively (https://www.fpbase.org/spectra). If an interaction exists between the target proteins, it will drive the fluorophores close enough for FRET to occur (Ahmed et al., 2019). FRET by fluorescence lifetime imaging (FRET‐FLIM) is an advanced imaging method that measures the decrease in lifetime—the average time that a fluorescent species remains in the excited state—of the donor molecule that occurs as a result of FRET (Becker, 2012). FRET‐FLIM is a more technically challenging method than spectral FRET, as it combines standard confocal laser scanning microscopy with features such as nanosecond pulsed lasers, two‐photon excitation with high‐repetition rate lasers in the red and near‐infrared light, and time‐correlated single‐photon counting (TCSPC), among other properties (Botchway et al., 2015; Kriechbaumer & Botchway, 2018; Schoberer & Botchway, 2014). In addition to TCSPC, FLIM can also be constructed around wide‐field imaging using gated image‐intensifying cameras that can be triggered on the subnanosecond time scale. FLIM instruments can also be built using frequency‐modulated light sources. Here, the intensity and frequency of the emitted fluorescence is measured and compared with that of the excitation light source. The difference is generally on the nanosecond time scale and provides a basis for another FLIM technique, frequency domain FLIM. Time‐gated and frequency‐domain FLIM do not provide as accurate or good spatial resolution as that provided by TCSPC‐confocal/multiphoton techniques. However, FRET‐FLIM offers several key advantages over spectral FRET. First, the fluorescence lifetime is a spectroscopic property that is largely independent of fluorophore concentrations, as opposed to intensity‐based methods. This can be easily accounted for by applying the Stern‐Volmer relationship. A Stern‐Volmer constant (Ksv) is obtained by using:

$$\frac{T_{0}}{T}\;=\;1\;+\;\mathrm{Ksv}(Q)$$

where Q is the “quencher” and τ are lifetimes in the presence and absence of quenching. Thus, FLIM is much more robust to variations in protein expression levels and to variations in the relative concentrations of donor and acceptor molecules (Bücherl, Bader, Westphal, Laptenok, & Borst, 2014; Godet & Mély, 2019).

Moreover, the technique is highly sensitive and does not present issues with spectral bleed‐through, as information is acquired solely from the donor molecule (Xing, Wallmeroth, Berendzen, & Grefen, 2016). Furthermore, the detector sensitivities vary depending on the wavelength being measured. Repeated excitation of the chromophores can also lead to photobleaching and photodegradation, which are rarely corrected for during imaging. However, FLIM‐based FRET does not suffer from these problems to the same extent. Photobleaching is also not a problem in lifetime measurements unless a new species or product is formed. However, it is not common for a new species to be formed from photodegradation that has identical excitation, emission, and lifetime as the donor molecule. Hence FRET‐FLIM offers several advantages over intensity‐based FRET. In addition, multiphoton microscopy has proven advantageous for imaging live biological samples. This technique uses mostly near‐infrared light with ultrashort (∼200 fs) and ultrafast (MHz) repetition rate lasers. This is particularly good for plant cell imaging where the use of light around 800 to 950 nm is not absorbed by the plant tissue. In multiphoton microscopy, only the molecules at the femtoliter focal volume are excited so that autofluorescence from the rest of the sample is eliminated. Furthermore, most detectors used for imaging are insensitive to the near‐infrared light, whereas the emission is anti‐Stoke shifted. Because of the inherit sectioning properties of multiphoton microscopy, no pinhole is required for a good 3D imaging.

FRET‐FLIM has been used to test protein‐protein interactions in a variety of biological systems ranging from mammalian cell lines (Day, Day, & Pavalko, 2021; Dikovskaya, Appleton, Bento‐Pereira, & Dinkova‐Kostova, 2019; Stubbs, Botchway, Slater, & Parker, 2005), plant tissue (Kriechbaumer et al., 2015; Kriechbaumer, Botchway, & Hawes, 2016; Liu et al., 2020; Long et al., 2018), and bacteria (Günther et al., 2019), as well as protein conformational change (Ahmed et al., 2019), among other properties. We describe here a method that tests FRET‐FLIM‐based protein‐protein interactions upon transient protein expression in tobacco leaf epidermal cells. We also describe how interaction studies can additionally provide data on protein membrane topology. This work showed the presence of protein‐protein interactions between recombinant subunits of the particulate methane monooxygenase (pMMO) enzyme complex and revealed new insights on membrane topology of one of the subunits.

## Basic Protocol 1 PROTEIN EXPRESSION IN TOBACCO LEAF EPIDERMAL CELLS VIA *AGROBACTERIUM*‐MEDIATED PLANT TRANSFORMATION

This protocol describes a method for the transient production of fluorescent proteins in tobacco leaf epidermal cells before FRET‐FLIM data acquisition. First, cell suspensions of *Agrobacteria* containing the genes of interest fused to fluorescence proteins (eGFP or mRFP) are generated. The cells are suspended in infiltration buffer, which contains the necessary components to promote plant tissue infection by *Agrobacteria*. The suspensions are introduced into tobacco leaves via infiltration. Infiltrated plants are incubated for 48 to 72 hr in standard tobacco growth conditions to allow protein expression. These leaf sections transiently expressing the proteins can be used for several applications, including FRET‐FLIM.

### Materials

*Agrobacteria* cultures transformed with proteins of interest (e.g., eGFP and mRFP)
Yeast extract beef (YEB) medium (see recipe)
Appropriate antibiotics
Infiltration buffer (see recipe)
4‐ to 6‐week‐old *Nicotiana tabacum* or *N. benthamiana* plants

15‐ml conical tubes (e.g., Thermo Fisher, cat. no 11889640)
28°C shaking incubator
2‐ml microcentrifuge tubes with safe lock (e.g., Eppendorf, cat. no. 0030121880)
Microcentrifuge (e.g., Sigma 1‐14 or equivalent)
Microvolume spectrophotometer (e.g., NanoDrop 2000 or equivalent)
1‐ml syringe, sterile (without needle)
Plant growth chamber

*CAUTION*: The bacteria used and the plants after infiltration are genetically modified (GM) material and have to be handled and disposed of according to GM regulations.

### Preparation of Agrobacteria cultures

1 Grow 3 ml overnight *Agrobacteria* cultures (e.g., RTN6‐GFP; Kriechbaumer et al., 2015) in 15‐ml conical tubes at 28°C with shaking at 200 rpm in YEB medium with the appropriate antibiotics encoded in the vector for constructs to be tested.
  Agrobacteria are usually transformed with one construct at a time; expression of multiple constructs in the same leaf can be achieved by mixing the appropriate Agrobacteria cultures.
  Antibiotics depend on the bacterial strain and the vectors used, but commonly used antibiotics are 25 mg/L rifampicin combined with 50 mg/L kanamycin or 50 mg/L spectinomycin. Store antibiotics for up to 1 week at 4°C or up to 12 months at –20°C for long‐term storage.
2 Transfer 1 ml of each culture into separate labeled 2‐ml microcentrifuge tubes.
3 Centrifuge 5 min at 2500 × *g*, room temperature, and remove supernatant.
4 Add 1 ml infiltration buffer, and gently resuspend pellet by pipetting, avoiding creating bubbles.
5 Centrifuge 5 min at 2500 × *g*, room temperature, and remove supernatant.
6 Resuspend pellet in 1 ml infiltration buffer.
7 Measure optical density at 600 nm (OD_600_) using a spectrophotometer.
8 Prepare various construct mixes (i.e., eGFP and mRFP protein fusion construct) in individual tubes, and dilute resuspension with infiltration medium until the required OD_600_ is reached.
  The optimum OD_600_ will vary according to the construct; thus different ODs have to be tested until sufficient expression levels are achieved. An OD_600_ of 0.1 is a good place to start with a new construct. After this, lower ODs (e.g., 0.03) or higher ODs (e.g., 0.2 to 0.3) can be tested.

### Tobacco leaf infiltration

**Figure 2:**
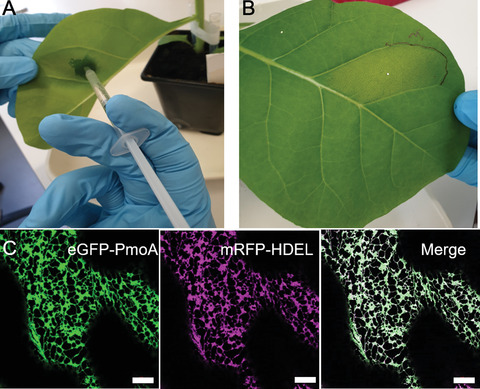
Tobacco leaf infiltration. (**A**) The *Agrobacterium* suspension is infiltrated into the leaf from the lower leaf side using a 1‐ml syringe. (**B**) The area filled with the *Agrobacterium* suspension is marked with a permanent pen. (**C**) Example confocal image for PmoA protein expression (green). Correct localization is shown by overlap with the endoplasmic reticulum marker mRFP‐HDEL (magenta; Brandizzi et al., 2003). Scale bars = 10 µm. PmoA, subunit A of the particulate methane monooxygenase enzyme complex.

9 For leaf infiltration, take up cells in a 1‐ml sterile syringe (without needle).
10 Using a 100‐μl pipette tip, punch a small hole on the abaxial side of the tobacco leaf, avoiding leaf veins.
11 Place syringe tip firmly against the underside of the leaf (abaxial) covering the hole, and press plunger down gently while exerting pressure against the other side of the leaf (adaxial) with your finger.
  The liquid will diffuse throughout the mesophyll air space (Fig. 2A).

12 Repeat for all constructs in at least two tobacco plants to allow for biological replicates.
13 Mark leaf upper epidermis with a permanent marker to keep track of transformation sites (Fig. 2B).
  Be aware that some marker ink will fluoresce on laser microscopes, so limit use of the permanent marker to the edge of the infiltrated region.
14 Place infiltrated plants back into growth conditions (21°C; 14 hr light, 10 hr dark; light intensity 120 μmol/m^2^/s; 50% humidity) for at least 30 hr to allow transformation and gene expression to happen.
  This growth period depends on the construct and protein longevity, but 40 to 48 hr are mostly required before fluorescence can be observed. Common starting points for testing are 48 and 72 hr after infiltration. Expression can be checked by confocal microscopy (Fig. 2C).
  Example data are shown in Figure 2.

## Basic Protocol 2 FRET‐FLIM DATA ACQUISITION AND ANALYSIS

**Figure 3:**
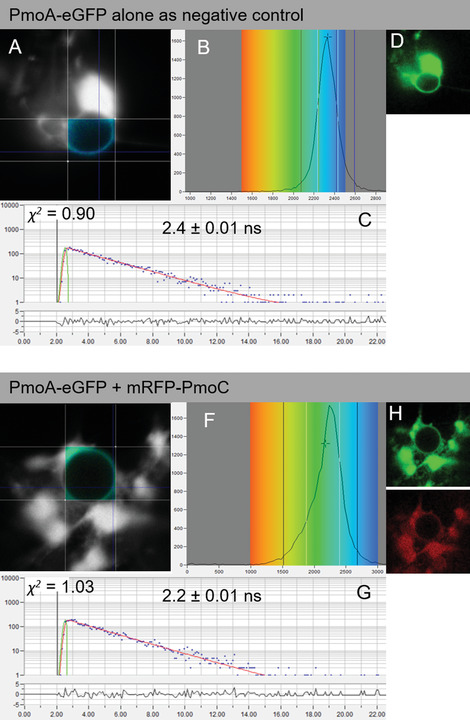
FRET‐FLIM analysis of PmoA‐eGFP without an interaction partner (**A**–**D**) or with mRFP‐PmoC (**E**–**H**). A and E display the FRET‐FLIM data, and the pseudocolored lifetime maps show the lifetime values for each point within the region of interest, whereas the distribution of lifetimes across the entire image is shown in B and F, with blue shades representing longer eGFP fluorescence lifetimes than green ones. C and G display representative decay curves of a single point with an optimal single exponential fit, where *χ^2^* values from 0.9 to 1.2 were considered an excellent fit to the data points (binning factor of 2). D and H are the respective confocal images for the analysis showing the eGFP construct in green and the mRFP construct in red. This example of FRET‐FLIM analysis shows that PmoA‐eGFP interacts with mRFP‐PmoC because the lifetime values for the eGFP/mRFP fusion pair (G; 2.2 ± 0.01 ns) are lower than those for the eGFP fusion alone (D; 2.4 ± 0.02 ns). eGFP, enhanced green fluorescent protein; FRET‐FLIM, Förster resonance energy transfer with fluorescence lifetime imaging microscopy; mRFP, monomeric red fluorescent protein; PmoA, subunit A of the particulate methane monooxygenase enzyme complex; PmoC, subunit C of the particulate methane monooxygenase enzyme complex.

To perform FRET‐FLIM experiments, a section (∼5 mm^2^) of transformed leaf expressing the proteins of interest is securely mounted onto a standard microscope slide with a coverslip and fastened down firmly with surgical tape. It is important to make sure there is enough water around the sectioned leaf piece before imaging. First, the eyepiece and white light is used to bring the leaf cells into focus. Second, the laser scanning system is engaged. Once protein expression is confirmed, the FRET‐FLIM system is activated. Using a pulsed light source, the same laser can be used for both confocal imaging and FLIM. Some FLIM systems use a continuous‐wave laser source for confocal imaging acquisition and a different pulsed‐laser source for FLIM acquisition. As of now, the FLIM software is different from the FLIM acquisition software as the two techniques are generally provided by two different manufacturers. This situation is slowly changing so that FLIM is becoming embedded in the same software such as within the Leica Falcon and Tau instruments that provide confocal imaging and FLIM simultaneously. The acquisition of FLIM images requires a fast timing device on the picosecond time scale. This is provided by the TCSPC device. A sensitive detector will measure the time between activation of the donor fluorophore and arrival of the emitted photon at the other detector. This process is repeated rapidly (MHz) during the pixel dwell time and over a period of ∼10 to 120 s depending on the brightness of the sample under observation. This is one of the drawbacks of FRET and FLIM in general, where very weakly emitting samples need tens of seconds for acquisition, and therefore any fast dynamics are lost. The measurements are used to build a FLIM image, which can be interrogated for evidence of interaction between donor and acceptor molecules (Fig. 3). Control datasets measuring the fluorescence lifetime determined when only a donor but no acceptor is present are compared with combinations of donor and acceptor. This ensures the collection of statistically significant data for interactions and can also inform membrane topology of the tested proteins.

### Materials

Infiltrated tobacco leaves (see Basic Protocol 1)
Scalpel
Microscope slides and coverslips
Two‐channel confocal and one‐ or two‐channel FLIM setup (e.g., Nikon TE2000‐U inverted microscope with modified Nikon EC2 confocal scanning system) with the following:
  Mode‐locked titanium sapphire laser (e.g., Coherent Lasers, Mira)
  Solid‐state, continuous‐wave, 532‐nm laser (e.g., Coherent Lasers, Verdi V18)
  High–numerical aperture objective (e.g., Nikon VC series)
  BG39 filter (e.g., Comar)
  633‐nm interference filter
TCSPC card (e.g., Becker and Hickl SPC 830 or SPC150 or equivalent)
FLIM image analysis software (e.g., Becker and Hickl SPCImage NG Data Analysis software, v5.1, or the latest version)

*CAUTION*: The detectors for FLIM are extremely sensitive, and care must be taken not to expose them to too much light. In our laboratory, all lights are switched off (using a switch on a remote control key fob), except for emergency lighting and luminescent guide strips for safety. The use of a remote light switch minimizes the user's movements in the darkened room.

### FLIM data acquisition

1 With a scalpel, cut out samples of infiltrated tobacco leaves (∼5 mm^2^ leaf disc, avoiding veins in the tobacco leaf and the injection hole). Place sample on a microscope slide, and cover with a coverslip. Place slide on a confocal/multiphoton FRET‐FLIM microscope system with the abaxial side facing the objective to collect confocal and FRET‐FLIM data (Schoberer & Botchway, 2014).
  For the experiments here, a two‐photon microscope built around a Nikon TE2000‐U inverted microscope is used with a modified Nikon EC2 confocal scanning system to enable near‐infrared laser wavelength (900 to 950 nm) for FLIM.
2 Set laser light at a wavelength of 920 nm using a mode‐locked titanium sapphire laser, with 200‐fs pulses at 76 MHz, pumped by a solid‐state continuous wave 532‐nm laser.
3 To illuminate specimens on the microscope stage, focus laser beam to a diffraction‐limited spot using a high–numerical aperture (1.2) objective such as water‐immersion objective.
4 Collect fluorescence emission without pinhole (non‐descan), bypassing the scanning system but instead passing through a BG39 filter to block the near‐infrared laser light.
5 Generate raw time‐correlated single‐photon data by using a TCSPC PC module SPC830. Analyze pixel‐by‐pixel TCSPC data to generate a FLIM image or map.
  Before FLIM data collection, eGFP and mRFP expression levels in the plant samples within the region of interest should be confirmed using a microscope with excitation at 488 and 543 nm, respectively (e.g., Nikon EC2 confocal microscope); emission for GFP and mRFP are at 520 ± 30 nm and 620 ± 40 nm, respectively.
6 Use a 633‐nm interference filter to reduce chlorophyll autofluorescence that will otherwise strongly obscure the mRFP and eGFP emissions.

### FLIM data analysis

**Figure 4:**
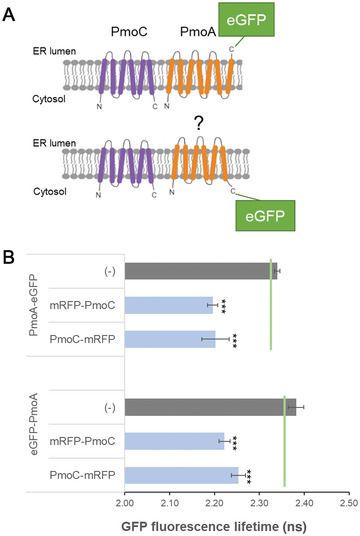
FRET‐FLIM interactions can resolve membrane topologies. (**A**) Schematic diagram of topology predictions for PmoC and PmoA. PmoC is predicted to feature six TMDs with both termini facing the cytosol. Predictions for PmoA differ between six and seven TMDs, and the C‐terminus could therefore either face the ER lumen (top) or the cytosol (bottom). (**B**) The bar graphs represent average fluorescence lifetimes (in ns) and the corresponding standard deviations for the eGFP donors PmoA‐eGFP and eGFP‐PmoA. The data show both donors with the two interaction candidates, mRFP‐PmoC and PmoC‐mRFP (blue bars), compared with PmoA‐eGFP and eGFP‐PmoA without interaction partners (grey bars). Lifetimes significantly lower than those of PmoA‐eGFP and eGFP‐PmoA alone (left side of the green line) indicate protein‐protein interactions. Significance was analyzed by Kruskal–Wallis test (*p < 0.05; *p < 0.01; ***p < 0.001); n = 4 with at least 5 technical replicates each. eGFP, enhanced green fluorescent protein; ER, endoplasmic reticulum; FRET‐FLIM, Förster resonance energy transfer with fluorescence lifetime imaging microscopy; mRFP, monomeric red fluorescent protein; PmoA, subunit A of the particulate methane monooxygenase enzyme complex; PmoC, subunit C of the particulate methane monooxygenase enzyme complex; TMD, transmembrane domain.

7 Analyze data by obtaining excited‐state lifetime values on a region of interest on the nucleus, and make calculations using analysis software.
8 Generate range of lifetime values (for each pixel) within a region of interest, and display as a distribution curve.
9 Fit decay data to a single exponential parameter as *f*(*t*) = *αε* ^−τ/τ^. To allow for optimal fit, consider only values with a χ*^2^* between 0.9 and 1.4 for statistical analysis.
  The intensity data provide the fluorescence decay function (f) at time t. Single‐, double‐, or triple‐exponential analysis of the decay yields the excited‐state lifetime (τ). The amplitude of the exponential components (a) defines the contribution to each lifetime component.
  χ^2^ describes the goodness of data fitted to the exponential function. Therefore, a value of 1 represents analysis that perfectly describes the decay data points.
10 Produce range of lifetimes per sample by considering the median lifetime and the minimum and maximum values for one‐quarter of the median lifetime values from the curve.
  The donor fluorophore alone without a potential interaction partner is used as negative control. Proteins known to interact (e.g., AtRTN1 with AtRTN1; Kriechbaumer et al., 2015; Sparkes et al., 2010) can be used as positive controls.
11 Analyze at least five cells from at least three independent biological samples (plants) per protein‐protein combination.
12 Take average and standard deviation of the ranges for interaction combinations and for negative and positive controls (Fig. 4).
  A reduction in lifetime of the donor fluorophore (eGFP) of >0.2 ns compared with the control is indicative of an existing protein‐protein interaction (Fig. 4; Table 1). It is also the case that a statistically significant lifetime reduction of 0.1 ns may indicate a long‐range protein‐protein interaction because of either the size of the proteins and the position of the GFP/RFP chromophores or a nondirect physical interaction. Two proteins separated from each other but close enough (within 10 to 12 nm) may show a small lifetime change in donor lifetime.
  Example data are shown in Figures 3 and 4 and Table 1.
  For information about sample data, see Understanding Results.

**Table 1:** Fluorescence Lifetimes in FRET‐FLIM Analysis^a^

Donor (eGFP)	Acceptor (mRFP)	Average eGFP fluorescence lifetime ± SD (ns)	Δ
PmoA‐eGFP	(–)	2.4 ± 0.01	–
PmoA‐eGFP	+mRFP‐PmoC	2.2 ± 0.01	0.2
PmoA‐eGFP	+PmoC‐mRFP	2.2 ± 0.03	0.2
eGFP‐PmoA	(–)	2.4 ± 0.02	–
eGFP‐PmoA	+mRFP‐PmoC	2.2 ± 0.01	0.2
eGFP‐PmoA	+PmoC‐mRFP	2.3 ± 0.02	0.1

eGFP, enhanced green fluorescent protein; FRET‐FLIM, Förster resonance energy transfer with fluorescence lifetime imaging microscopy; mRFP, monomeric red fluorescent protein; PmoA, subunit A of the particulate methane monooxygenase enzyme complex; PmoC, subunit C of the particulate methane monooxygenase enzyme complex; SD, standard deviation.

^a^ Interactions between PmoA‐eGFP and eGFP‐PmoA with mRFP‐PmoC and PmoC‐mRFP were analyzed. Donor and acceptor protein constructs are listed together with the average fluorescence lifetime (in ns) for the donor fluorophore and the SD for each combination. The difference between control and test samples was calculated (Δ). It was previously reported that a reduction in excited‐state lifetime of 0.2 ns is indicative of energy transfer (Stubbs et al., 2005). For each combination, at least four biological samples with a minimum of five technical replicates were used for analysis. Negative (PmoA‐GFP, GFP‐PmoA) controls are included.

## REAGENTS AND SOLUTIONS

### YEB medium

5 g/L beef extract
1 g/L yeast extract
5 g/L sucrose
0.5 g/L MgSO_4_·7H_2_O
Deionized water
Sterilize by autoclaving
Store at room temperature for up to 12 months

### Infiltration buffer

50 mM 2‐(*N*‐morpholino)ethanesulfonic acid (MES)
2 mM Na_3_PO_4_·12H_2_O
0.1 mM acetosyringone
5 g/L glucose
Deionized water
Prepare fresh the day before use
  Prepare MES and Na_3_PO_4_·12H_2_O stocks in advance, and store at 4°C for up to 1 month. Store acetosyringone stock at –20°C for up to 12 months. Filter sterilization is not required.

## COMMENTARY

### Background Information

FRET‐FLIM is a state‐of‐the art method to measure protein interactions. Proteins in close proximity (ideally molecular distances of 1 to 10 nm), and therefore most likely to physically interact, have energy transfer processes described as Förster or fluorescence resonance energy transfer (FRET). This method was described by Theodor Förster over 70 years ago (Förster, 1948) and is based on the energy transfer from an excited fluorescent molecule (donor) to another nonexcited fluorescent molecule (acceptor) in close proximity. FRET will only occur if the donor emission spectrum overlaps with the acceptor absorption spectrum. During FRET, the decay rate is reduced because of quenching that depletes the excited state of the donor fluorophore and leads to shortening of the donor fluorescence lifetime. By measuring changes in the excited‐state lifetime of the donor for each pixel in the image, steady‐state FRET is improved. This is described as FRET‐FLIM. FRET‐FLIM in general is advantageous over FRET as, for example, it is independent of local fluorophore concentration or wavelength‐dependent light scattering. In addition to the analysis of protein‐protein interactions and within the process of data acquisition for such, FRET‐FLIM also allows for determining the topology of proteins in the membrane and their subcellular localization.

### Critical Parameters

For adequate measurements, sufficient expression levels of both donor and acceptor proteins in the plant cells are required. These can be optimized by varying the OD_600_ of the *Agrobacterium* cultures and the time after infiltration and before visualization. A good starting point is an OD_600_ of 0.1 and visualization 72 hr after infiltration.

FRET‐FLIM measurements and fluorescence lifetimes are dependent on the cellular surrounding and the localization of the proteins tested. Hence control proteins should be localized to the same part of the cell as the proteins of interest. Best measurement results for the endoplasmic reticulum (ER) have been shown for the nuclear envelope because the ER here is less mobile, which allows for the time required for capturing FRET‐FLIM data. Latrunculin B (Gibbon, Kovar, & Staiger, 1999) can be applied to the leaf discs to depolymerize the actin cytoskeleton and therefore inhibit movement of the ER. FRET‐FLIM measurements can also be taken on ER cisternae after application of latrunculin B. Here it should be considered that latrunculin B is changing the ER structure and induces cisternae.

Correct selection of fluorophore pairs for FRET‐FLIM is important. One common issue in FRET‐FLIM occurs when there is large spectral overlap of the fluorophore pairs chosen for imaging. Issues can arise when fluorescence from the donor molecule spreads into the acceptor channel and when the acceptor is excited by the laser exciting the donor (Fig. 1). Recommended FRET‐FLIM fluorophore pairs for plants include eGFP and mRFP (Kriechbaumer et al., 2015) or mCherry, T‐sapphire and mOrange (Denay, Schultz, Hänsch, Weidtkamp‐Peters, & Simon, 2019), SYFP2 and mRFP, and SCFP3A and SYRP2 (Long et al., 2018).

### Troubleshooting

Possible problems and troubleshooting suggestions are provided in Table 2.

**Table 2:** Troubleshooting Guide for Protein Expression in Tobacco Leaves

Problem	Possible cause	Solution
Low fluorescence levels	Too low protein expression levels	Optimize expression protocol (*Agrobacterium* OD, time after infiltration before visualization); increase laser excitation average power
Necrosis and damage to plant leaves	Expressed proteins have toxic effect or overexpression inhibits plant physiology	Optimize expression protocol and reduce expression levels (*Agrobacterium* OD, time after infiltration before visualization)
False negative interactions due to fluorescent tag position	Fluorophores are not located sufficiently close to each other for FRET to occur leading to false negative results	Consider placing fluorescent tags at each end of the protein of interest
Crosstalk between donor and acceptor	Leaking of donor fluorescence into the acceptor channel and/or acceptor excitation by the donor excitation laser	Optimize fluorophore pairings so they are spectrally well separated (e.g., commonly used donor/acceptor fluorophore pairs: mRFP and eGFP); consider replacing fluorescence filter of the donor channel

eGFP, enhanced green fluorescent protein; FRET, Förster resonance energy transfer; mRFP, monomeric red fluorescent protein; OD, optical density.

### Understanding Results

FRET‐FLIM analysis provides fluorescence lifetime values for each point within the region of interest (Fig. 3A,E), allowing for an overall lifetime or distribution value for each image. The donor (eGFP) protein alone is generally used as the main control. A negative control with a protein combination known not to interact can be an advantage. Proteins known to interact should be used as positive controls where possible. Alternatively, synthetic donor‐receptor fluorophore fusions targeted to the same cellular localization as the proteins of interest can be used. The lifetimes are dependent on various parameters such as the type of donor fluorophore (e.g., eGFP or CLOVER; Lam et al., 2012), the cellular environment, and localization of the fluorophores. Hence, these parameters need to be kept constant.

For negative and positive controls and each donor‐receptor combination, average and standard deviation for the lifetimes for all biological and technical replicates are calculated (Fig. 4B). A reduction in excited‐state lifetime of 0.2 ns or more is an excellent indicator of energy transfer (Stubbs et al., 2005). For statistical significance Kruskal‐Wallis analysis can be applied (Tilsner & Kriechbaumer, 2022). Significant differences in lifetime between donor alone and donor‐receptor combinations can indicate more transient protein‐protein interactions or those occurring at larger distances. Data are best presented as bar graphs (Fig. 4B) or alternatively in a table (Table 1).

The acquired data can also yield additional information on protein membrane topology. In this example, subunit C of the pMMO enzyme complex (PmoC) is predicted to have six transmembrane domains (TMDs) spanning the ER membrane with both termini facing the cytosol (Fig. 4A; http://cctop.ttk.hu/). However, the membrane topology for subunit A of the pMMO enzyme complex (PmoA) was unclear, and predicted protein structures varied between six or seven TMDs (Fig. 4A). This would result in the C‐terminus of PmoA facing either the ER lumen or the cytosol (Fig. 4A). Because a fluorescent tag fused to the C‐terminus of PmoA (PmoA‐eGFP) can interact with both mRFP‐PmoC and PmoC‐mRFP (Fig. 4B; Table 1), both termini of PmoA face the cytosol, and therefore the six‐TMD model for PmoA is likely. Of course, even if topology data are not part of the experimental question, membrane topology and localization of the proteins to be tested need to be taken into account because physical interactions and, with that, changes in fluorophore lifetimes are not possible via membrane barriers or in different physical localizations.

### Time Considerations

Initial preparation of constructs with appropriate fluorophore combinations will take a variable amount of time, depending on the method of cloning used and the number of combinations required. In addition, optimizing the OD of the infiltration medium and the required wait period after infiltration before imaging may take several weeks (depending on the specific proteins).

Once reliable expression can be assured, the entire process of infiltration to preforming FRET‐FLIM can be completed in a week. Approximately 1 hr is required to prepare the necessary *Agrobacterium* cultures, which are then grown overnight. The following day, *Agrobacterium*‐mediated transformation can be performed, with practice and depending on the number of combinations required, within 2 to 3 hr. The plant is then left for 2 to 3 days, requiring little input except for watering.

After this period has elapsed, with practice, 50 to 75 FRET‐FLIM images can be generated in a day. This corresponds to about three donor‐acceptor combinations with the corresponding negative controls (10 cells per combination from at least two different plants for n = 2).

### Author Contributions

**Tatiana Spatola Rossi**: Data curation, investigation, resources, visualization, writing–review and editing; **Charlotte Pain**: Investigation, visualization, writing–review and editing; **Stanley W. Botchway**: Conceptualization, formal analysis, resources, supervision, visualization, writing–review and editing; **Verena Kriechbaumer**: Conceptualization, formal analysis, funding acquisition, investigation, project administration, resources, supervision, validation, visualization, writing–original draft.

### Conflict of Interest

The authors declare no conflicts of interest.

## Data Availability

The data, tools, and material (or their source) that support the protocol are available from the corresponding author upon reasonable request.
